# Advances in Genome Editing for Plant Disease Resistance Breeding

**DOI:** 10.3390/plants15111644

**Published:** 2026-05-27

**Authors:** Ciro Gianmaria Amoroso, Giuseppe Andolfo

**Affiliations:** Department of Agricultural Sciences, University of Naples ‘Federico II’, 80055 Portici, Italy

**Keywords:** genome editing, precise editing, plant diseases, biotic stress, plant breeding

## Abstract

Plant diseases remain a major constraint to crop productivity and global food security. Conventional breeding has long been used to develop resistant cultivars through the introgression of resistance traits from wild relatives and the selection of favorable phenotypes. However, this process is often slow and limited by linkage drag, known genetic diversity, intrinsic genetic limitations, and the rapid evolution of pathogen populations. Molecular breeding strategies, including marker-assisted selection and genomic selection, have improved the precision of resistance breeding but still rely on existing genetic variation. Recent advances in genome editing technologies are transforming plant breeding by enabling precise modification of gene targets. CRISPR-based systems allow targeted gene knockouts, promoter editing, allelic replacement, and multiplex editing to rapidly generate resistance traits. Many studies have demonstrated that editing susceptibility genes or regulatory regions can enhance resistance to diverse pathogens. Recent research shows that resistance can also be improved by targeting non-classical genes involved in plant immunity, including transcription factors, membrane transporters, heat shock proteins, cell wall-related genes, metabolic enzymes, and epigenetic regulators. Emerging tools such as base editing, prime editing, regulatory tools, and transposon-associated genome engineering systems are further expanding the precision and versatility of plant genome editing. Despite these advances, challenges related to delivery systems, editing efficiency, regulatory frameworks, and field validation remain. Continued technological progress and improved knowledge of plant immune networks will be essential to fully integrate genome editing into crop improvement programs.

## 1. Introduction

Crops rarely face a single stress. Environmental changes are increasing the likelihood that cultivated species are subject to multiple stresses in sequence or in combination, drastically reducing performance and yield [1,2]. One of the main constraints under combined stresses is that plant behavior cannot be explained as the simple sum of individual stress effects. Studies have evidenced that under multiple-stress conditions, the responses are unique and differ from those observed in single-stress experiments [3]. Therefore, breeding strategies optimized for single-stress scenarios can fail under field conditions [4]. So far, conventional breeding has been the most widely used strategy to protect crops from diseases. This approach starts with the identification of resistance sources in wild species, landraces, or related germplasms. Thereafter, breeders attempt to introduce the identified resistance traits in cultivated species through crossing and backcrossing steps. Finally, progeny that shows desirable resistance under controlled inoculation is selected [5]. Historically, conventional breeding has been flanked by molecular approaches that improved its precision and efficiency. Techniques such as marker-assisted selection (MAS) and quantitative trait loci (QTL) mapping have traditionally played critical roles in identifying and selecting desirable resistance traits [6,7]. In addition, genomic selection recently emerged as a promising tool to predict plant performance based on its genomic information [8]. However, new emerging techniques, particularly genome editing (GE), are bringing plant breeding to a new dimension for the rapid generation of resistance traits without the extensive backcrossing required in conventional breeding [2]. Plant disease resistance is often associated with resistance (R) genes, which can encode receptor-like kinases, receptor-like proteins, or nucleotide-binding leucine-rich repeat (NB-LRR) proteins, and can activate strong, race-specific defense responses [9]. Recent studies pointed out that the resistance is not governed by a single genetic component but rather by a network of immune receptors [10]. Moreover, a growing number of works demonstrated that plant resistance extends well beyond canonical R-gene families and can also be achieved by manipulating non-R genes directly [11]. Membrane transporters, metabolic enzymes, hormonal pathways, transcription factors (TFs), heat shock proteins (HSPs), and cell wall components constitute a significant group of non-R gene targets, as they are consistently induced by biotic stress and are regulated at multiple levels [11,12]. In this review, we provide a progressive overview of strategies used to improve plant disease resistance, from classical breeding to the most recent precision breeding technologies. Here, we focus on GE technologies, highlighting well-established CRISPR-based tools and emerging systems. Subsequently, different genetic targets beyond classical R-genes are discussed. Finally, technical and regulatory challenges are examined, and future perspectives for precision breeding in crop improvement are outlined.

## 2. Conventional Breeding for Resistance

In plants, natural hybridization is acknowledged as a crucial evolutionary process [13]. For years, conventional breeding for pathogen resistance attempted to replicate this method. The identification of resistant germplasm is often carried out from wild relatives or traditional cultivars and is followed by systematic crossing with elite varieties to introgress disease resistance traits (Table 1). Using this strategy, substantial progress has been achieved, resulting in cultivars resistant to some of the most economically damaging plant pathogens [14,15]. Several studies documented the effectiveness of conventional breeding in generating disease-resistant plants, in some cases providing stable protection against major pathogens over multiple decades [16]. For example, in cereals, resistance breeding strategies led to the identification of resistance traits to counteract the emergence of highly virulent pathogens such as the fungus *Puccinia graminis* [17,18]. Similarly, in rice, the transfer of resistance traits from wild or resistant germplasm into elite varieties has played a key role in controlling devastating diseases and securing crop productivity [19]. Additionally, conventional breeding has successfully produced disease-resistant potato cultivars primarily through the crossing of cultivated varieties with wild relatives [20]. However, while resistance based on a single gene is effective in the short term, successful long-term resistance requires genetic complexity at multiple levels [21]. One of the most pressing limitations in conventional breeding is the long breeding cycles required to establish a new resistant variety (Table 1). This delay can be particularly problematic when pathogen populations evolve rapidly [2]. Furthermore, the phenomenon known as linkage drag, where beneficial resistance traits are inherited along with undesirable genetic material from the donor parent, can complicate the breeding process, potentially leading to the introgression of traits that are not agronomically desirable and may even reduce the overall crop performance [22]. Last but not least, the obtained varieties rely on a limited genetic diversity within populations. As a result, cultivated species often possess a narrowed immune network [23], becoming more susceptible to pathogen adaptation. Since many diseases can quickly overcome single R-genes, the durability of these defenses is often compromised in the field [24]. Because of these limitations, faster and more precise approaches to breeding disease-resistant crops have been developed. The integration of genomic tools and molecular breeding techniques offered parallel techniques to enhance efficiency and precision for resistance breeding [25].

## 3. Early Precision Breeding Approaches

We could say that an early precision breeding for plant disease resistance emerged when breeders supported conventional breeding with molecular information to guide selection (Table 1). Different authors reviewed the successes of disease resistance selection traits guided by molecular information [26,27]. For instance, a well-known success of MAS is the development of bacterial blight-resistant rice through marker-assisted backcrossing. Using molecular markers to combine R-genes into an elite variety, Sundaram et al. [28] generated highly resistant rice lines. MAS has also been used to combine multiple QTLs involved in pathogen interactions. In wheat, MAS enabled the pyramiding of major resistance QTLs for Fusarium head blight resistance, facilitating the combination of multiple loci into elite varieties [29]. Despite these advances, MAS approaches remain constrained by existing genetic variation and by the time required for repeated crossing and selection [7]. More recently, genomic selection has further increased the precision and efficiency of resistance breeding by using genome-wide information to predict complex traits controlled by multiple loci [30,31]. However, this approach has important limitations, including differences in marker effects across subpopulations and the risk of “double counting” population structure in prediction models [27]. Moreover, limited phenotyping throughput and accuracy remain a major bottleneck for accurately linking genotype to phenotype [27]. In addition to molecular markers and genomic approaches, early transgenic strategies were developed to directly overexpress or silence specific genes involved in disease interactions, providing a more targeted route to the modification of specific traits [32] (Table 1). The transgenic approach can be used where conventional cross-breeding is not applicable. Hundreds of papers have appeared in the academic literature that describe genes that have been introduced or manipulated by transgenic techniques to make host plants more resistant to one or multiple diseases [33]. While gene overexpression has long been investigated, here we provided various studies that have been released in the last three years, which have reported that transgenic overexpression of specific disease-related genes can significantly enhance resistance to a broad range of pathogens across different economically important plant species. For example, the transgenic overexpression of tomato thaumatin-like proteins *SlTLP5* and *SlTLP6* increased resistance to multiple soil-borne diseases by increasing β-1,3-glucanase activity [34]. A second example in tomato comes from the overexpression of the *BROAD-SPECTRUM RESISTANCE 1* (*BSR1*) gene, which enhanced resistance to the bacteria *Pseudomonas syringae pv. tomato DC3000* and the fungus *R. solani* [35]. Similarly, the transgenic overexpression of the TF *AbMYB11* in Arabidopsis significantly increased the total accumulation of phenolics and flavonoids and improved resistance to *P. tolaasii* [36]. In rice, Shen et al. [37] conducted a study overexpressing the β-1,6-glucanase (GluM) gene from myxobacteria, reporting a conferred high resistance to three major rice diseases: rice blast, sheath blight, and false smut. Similarly, Huang et al. [38] reported that the overexpression of the *Atp2* gene enhanced blast disease resistance. Experiments conducted in other economically relevant crops, such as wheat, showed that the overexpression of *TaCERK1* and *AtCERK1* confers resistance to multiple fungal diseases, including Fusarium head blight, stripe rust, and powdery mildew [39]. As recently reviewed, the transgenic overexpression of specific disease-related genes in wheat has been shown to enhance resistance against multiple pathogens. For example, wheat lines engineered to overexpress pathogenesis-related protein genes displayed increased resistance to both leaf rust and stripe rust [40]. In potato, Jacobs et al. [41] showed that the overexpression of the *GSL1* gene prompted plant defense against pathogens. However, the overexpression of genes involved in plant disease resistance can result in cellular damage, particularly through the induction of cell death, and a general plant fitness reduction [39]. To overcome these issues, other transgenic approaches have been used. For example, RNA interference (RNAi) is used to transiently transform plants to enhance disease resistance. A recent study by Al-Roshdi et al. [42] used an RNAi-based construct to confer tomato resistance against the tomato yellow leaf curl virus (TYLCV), targeting the TYLCV replicase gene. A second typology of experiments focuses on the tomato RNA silencing machinery itself. Indeed, Zhang et al. [43] reported that blocking of tomato miR6026, involved in the regulation of *SlDCL2b*, can enhance resistance to potato spindle tuber viroid (PSTVd) infection. Interestingly, a recent work of Xie et al. [44] reported the generation of transgenic rice expressing RNAi constructs targeting viral gene fragments. These rice lines were marker-free and showed enhanced resistance to rice ragged stunt virus (RRSV) and rice grassy stunt virus (RGSV). Similarly, Wang et al. [45] used RNAi to suppress disease caused by *Rhizoctonia solani* in *Nicotiana benthamiana* by silencing endoPGs or *RPMK1* in host plants. A specific application of RNAi that uses engineered viral vectors to trigger gene silencing is the Virus-Induced Gene Silencing (VIGS), primarily used for functional genomics studies, this technique has been successfully applied to induce targeted gene silencing in several host plants [46]. However, despite these transgenic approaches being useful to validate gene function and improve plant disease resistance, breeding programs have continued to rely on non-transgenic management strategies. This is mainly due to regulatory constraints and limited public acceptance of genetically modified crops in many regions [47]. In addition, the long-term durability of resistance traits remains difficult to guarantee, as pathogen and pest populations can rapidly evolve and overcome established resistance mechanisms [48]. Finally, the rapid emergence of modern GE technologies has provided a more precise, faster, and cost-effective alternative to classical transgenics, further limiting the field deployment of earlier transgenic approaches [49] (Table 1).

## 4. Genome Editing as a Tool for Precision Breeding for Disease Resistance

Different from early transgenic strategies that require an integrated cassette to overexpress exogenous genes or RNA silencing mechanisms, GE emerged for the precise modification of endogenous plant genes. By enabling targeted changes at specific genomic loci, GE techniques overcome several limitations of classical transgenics. Zinc-finger nucleases (ZFNs) and transcription activator-like effector nucleases (TALENs) represent the first generation of programmable GE technologies. In these systems, engineered DNA-binding proteins are fused to endonuclease domains to induce site-specific DNA double-strand breaks, enabling targeted genome modification [50]. Although ZFNs and TALENs demonstrated the feasibility of GE and have been applied in plant research, their complexity, cost, and limited scalability constrained widespread adoption in breeding programs [51]. Similarly, meganucleases are also included in the first generation of sequence-specific GE tools, recognizing long DNA motifs with high specificity, but their intrinsic properties, such as the binding and cleavage domain that overlap with each other, the lack of a modular DNA-binding domain architecture that are present in ZFNs and TALENs, and in some cases, the increase in off-target sites, have restricted their application in plant breeding [52]. Therefore, we could state that the advent of CRISPR-Cas9 technology marked the beginning of GE as a broadly applicable and precision breeding technology in plants. Indeed, so far, CRISPR-Cas9 has been established as the most widely used GE platform for precise resistance breeding because it allows precise targeting of specific genomic loci in a fast and cost-saving manner [53]. This technique is increasingly regarded as a powerful approach to accelerate the development of disease-resistant cultivars [54]. The system consists of a programmable single-guide RNA (sgRNA) that directs the Cas9 nuclease to a complementary DNA sequence adjacent to a protospacer-adjacent motif (PAM). Once bound, Cas9 introduces a double-strand break at the target site. In plants, these breaks are predominantly repaired by error-prone repair pathways, such as non-homologous end joining (NHEJ), which frequently generate small insertions or deletions at the cut site [55]. These mutations can disrupt gene function, resulting in stable loss-of-function alleles that are useful for disabling susceptibility genes (S-genes) and enhancing disease resistance. Several studies reviewed canonical examples [56,57,58]. Among the most recent applications, we could mention tetra-allelic deletion mutants generated in potato that confer resistance to late blight [59] or the inhibition of the *StERF3* gene by dual targeting that enhances resistance to late blight [60]. Moreover, the editing of the host translation initiation factor *eIF4E1* in potato was reported to extend the resistance spectrum to potato virus Y (PVY) [61]. Similarly, the inhibition of S-genes in other horticultural plants allowed the obtainment of enhanced resistance to specific pathogens [58]. Beyond single-gene targeting, CRISPR-Cas9 has been further developed to enable simultaneous editing of two or more genomic sites through multiplexing strategies, allowing precise modification of complex traits [62]. For example. Tran et al. [63] used different gRNAs that resulted in the precise elimination of *HyPRPI* functional domains, generating multi-stress-tolerant alleles. Multiplexing can also be used to target multiple genes simultaneously. For instance, a recent study generated stable tomato lines with enhanced resistance to Fusarium wilt disease by knocking down both *XSP10* and *SlSAMT* genes [64]. However, while Cas9-mediated knockouts could be effective for generating resistance through gene disruption, the potential off-target occurrence can limit the application of this technology [58]. Moreover, not all resistance traits can be achieved via loss-of-function alleles. The CRISPR-Cas system has also been used to exploit homology-directed repair (HDR) that enables site-specific transgene insertion using a donor template. A practical example was provided in rice, where CRISPR-Cas9 was used to mediate HDR and obtain resistance to bacterial blight by adding a specific effector binding element (EBE) (*EBEAvrXa23*) in the gene promoter region, with an efficiency of around 1.8% [65]. In parallel, other Cas variants have also been used to mediate HDR in plants, including Cpf1/Cas12a with an increase in efficiency up to 8% [66]. Despite this, HDR efficiency remains very low, limiting its practical application for precise trait engineering [58]. In parallel, base editing (BE) and prime editing (PE) emerged as important innovations that enable precise nucleotide substitutions or sequence changes without relying on double-strand breaks [54]. An experimental example of the use of the PE system to enhance plant disease resistance, is provided by Gupta et al. [67] in rice. In this study, the authors could efficiently knock-in a TAL effector binding element into the promoter of a dysfunctional resistance gene, creating a transcription activator-like effector (TALE) nucleases-inducible defense allele [67]. Furthermore, they edited the susceptibility-associated TF gene *TFIIAγ5* to recreate a resistance variant and obtained a broad-spectrum resistance to bacterial blight caused by *Xanthomonas oryzae pv. oryzae*. Similarly, CRISPR-Cas9-based cytidine (CBE) and adenine (ABE) base editors have been used to alter the EBE in the promoter of the *SWEET14* susceptibility gene, reducing pathogen activation and conferring resistance to African *Xanthomonas oryzae* in rice, without detectable off-target mutations or major agronomic penalties [68]. Recent advancements in BE have expanded beyond simple A/T to G/C or C/G to T/A conversions. These systems use a DNA glycosylase or a related base-excision-repair enzyme module to expand the range of base substitutions beyond standard CBE and ABE editors. For example, Li et al. [69] developed an A-to-K BE system that enables efficient and heritable A-to-G, A-to-T, and A-to-C substitutions in both rice and tomato. However, despite its broad usage, CRISPR-Cas9 editing for plant disease resistance still faces technical constraints due to editing efficiency and persistent concerns about off-target effects [70]. To address these limitations, a growing number of bioinformatic tools have been developed to optimize guide RNA design and reduce off-target activity [58]. Moreover, BE has limitations in its range of base conversions, while PE efficiency and accuracy are still far from satisfactory [71,72].

## 5. Recent Precision Breeding Approaches and Emerging Technologies

While CRISPR-Cas9, BE, and PE broadened the use of GE in plant precision breeding, ongoing research continues to expand the available toolboxes. In recent years, several CRISPR-based systems and alternative programmable nucleases have been engineered to overcome current technical limitations [73]. While the widely used SpCas9 can be used to target NGG PAMs, it has low tolerance for different PAMs, which restrains the application of this technology. Therefore, to overcome these limitations, highly flexible PAMs SpCas9, such as SpCas9-NG, xCas9, XNG-Cas9, or SpRY, have been used in crops like rice and tomato, and in Arabidopsis as a model plant [74] (Table 2). The engineering of novel Cas9 orthologs and variants is offering new opportunities for enhancing precision, multiplex editing, and targeted insertion [75], broadening the field of CRISPR-Cas application in plants. For example, the Cas12 (Cpf1/Cas12a) has been positioned as a key complement to SpCas9 because it offers a PAM targeting T-rich regions (TTTV), offering new alternatives for research and breeding [76]. So far, relevant applications of Cas12a for enhancing plant disease resistance have been reported, including the utilization of Cas12a to target different ORFs of the cotton leaf curl multan virus (CLCuMuV) genome at multiple sites simultaneously, eliminating the disease symptoms in *Nicotiana benthamiana* and *Nicotiana tabacum* [77]. However, Cas variants not only differ for the target PAM sequence but also for the transport mechanisms [78]. For example, the crRNA used by the Cas12a is around 43 nt, which is significantly shorter than the sgRNA of SpCas9 (~100 nt) [79]. The identification and engineering of novel Cas variants aim to expand the targetable DNA sequences. Recently, more Cas12a-type variants have been generated in plants, such as the Mb2Cas12a-RVRR (5′-TTTV, VTTV, TATV, TYCV, CCCV, CTCV-3′ PAMs) [80] (Table 2). Furthermore, it is known that GE efficiency is reduced under low-temperature conditions [81]. For this purpose, temperature-tolerant Cas12a (ttCas12a) nucleases were engineered from LbCas12a and optimized for plant GE. A recent research reported an efficient use of PAM-relaxed, temperature-tolerant Mb3Cas12a (ttMb3Cas12a) for rice, maize, and tomato editing [82]. In addition to DNA-targeting editing systems (Table 2), RNA-guided RNA nucleases such as Cas13 have been applied in plants for sequence-specific RNA targeting. Cas13 systems such as Cas13a, Cas13b, and Cas13d can reduce viral RNA accumulation and enable transcript knockdown without altering the plant genome, offering a complementary strategy for RNA virus resistance [83]. In parallel, the continuous discovery of new CRISPR effectors has further expanded the GE toolbox. Among these, Cas14 has emerged as a miniature CRISPR nuclease capable of targeting single-stranded DNA (ssDNA) without the need for the PAM sequence but requires tracrRNA and the crRNA, similarly to Cas9 protein [84]. This feature makes Cas14 particularly attractive for potential applications against plant ssDNA viruses; however, current evidence is largely limited to mechanistic and proof-of-concept studies, and its application in plant GE and disease resistance breeding remains to be proved.

Although a wide diversity of Cas orthologs has been identified, only a limited set of CRISPR systems is currently highly efficient and widely usable for GE-mediated plant disease resistance, although ongoing research is rapidly expanding this repertoire. Beyond the identification of new Cas variants and novel size-reduced nucleases, recent studies have also introduced different RNA-guided systems that can extend the application of GE in plants. Among these emerging approaches, CRISPR-based transcriptional regulators such as CRISPR activation (CRISPRa) and CRISPR interference (CRISPRi) have been developed to modulate gene expression without altering the DNA sequence. These systems rely on catalytically inactive Cas proteins fused to transcriptional activators or repressors to regulate endogenous genes expression in a targeted manner. For instance, CRISPRa was used to trigger the activation of *SlPR*-1 and *SlPAL2* in tomato enhancing resistance to bacterial pathogens by strengthening defense responses and processes related to cell wall [85,86]. In contrast, CRISPRi enables targeted repression of gene expression by directing dCas proteins to promoter regions and allowing the downregulation of S-genes without modifying DNA sequence, which makes it particularly attractive to reduce pleiotropic effects associated with DNA modifications. A recent study in cassava demonstrated that a specific system of dCas9 named SunTag, mediated repression of the S-genes *nCBP*-1 and *nCBP-2*, reducing susceptibility to cassava brown streak disease [87]. In parallel, a transcriptional enhancer for genes (CRISPRe), has been developed to improve the expression of heterologous and endogenous genes and the biosynthesis of products by facilitating transcriptional elongation [88]. Together, these approaches expand the CRISPR toolbox well beyond sequence modification and provide new opportunities for precision breeding.

**Table 2:** Cas9 and Cas12 variants used for DNA targeting in plant genome editing and their relative PAMs.

Cas-Variant	PAM	Application	Maturity	Reference
SpCas9	NGG	DR	Widely adopted	[89]
SpCas9-NG	Broad NG PAMs	GE	Established	[90]
xCas9	NG, GAA, and GAT	GE	Emerging	[91]
XNG-Cas9	NG relaxed	GE	Emerging	[92]
SpCas9-VQR	NGAN–NGNG	GE	Emerging	[93]
SpCas9-EQR	NGAG	GE	Emerging	[93]
SpCas9-VRER	NGCG	GE	Emerging	[93]
SpCas9-NRRH	NRRH	GE	Emerging	[94]
SpCas9-NRCH	NRCH	GE	Emerging	[94]
SpCas9-NRTH	NRTH	GE	Emerging	[94]
SaCas9	NNGRRT	GE	Established	[95]
SaCas9-KKH	NNNRRT	GE	Emerging	[95]
St1Cas9	NNAGAA–NNGGAA	GE	Emerging	[96]
SpG	NG	GE	Emerging	[97]
SpRY	NRN (preferred) and NYN (tolerated)	GE	Established	[98]
iSpyMacCas9	NAAR	GE	Emerging	[99]
ScCas9	NAG	GE	Emerging	[100]
LbCas12a	TTTV	DR	Widely adopted	[101]
AsCas12a	TTTV	GE	Established	[101]
LbCas12a-RRV	TTTV (relaxed)	GE	Emerging	[102]
Ev1Cas12a	TTTV–VTTV	GE	Emerging	[103]
Hs1Cas12a	TTTV–VTTV	GE	Emerging	[103]
Mb3Cas12a	TTTV and TTV (relaxed)	GE	Emerging	[104]
Mb3Cas12a-R	TTV (relaxed)	GE	Emerging	[81]
Mb3Cas12a-RRR	TTV (relaxed)	GE	Emerging	[81]
AaCas12b	VTTV/TTN	GE	Established	[105]
AacCas12b	VTTV/TTN	GE	Emerging	[105]
BthCas12b	VTTV/TTN	GE	Emerging	[105]

Application: DR, demonstrated in plant disease-resistance applications; GE, experimentally validated in plant genome editing but not clearly demonstrated in disease-resistance applications. Maturity: widely adopted, broadly used in plant genome editing and/or disease-resistance studies; established, validated in multiple plant systems but less routinely used than standard SpCas9; emerging, recently developed, specialized, or less broadly adopted.

In parallel with the development of new CRISPR systems and engineering of Cas variants for plant GE, BE and PE platforms have also been optimized to improve editing efficiency and precision. For instance, optimization of ABEs in rice showed that changes in editor architecture can markedly increase conversion efficiency, with maximum efficiencies approaching 96% [106]. Further improvements were obtained by Wang et al. [107], who combined optimized core components with refined nuclear localization signals and developed a multiplex super-assembled ABE system with higher base-editing efficiency in rice. In wheat and maize, systematic optimization of Cas12a base editors increased activity from almost undetectable levels to robust editing and produced base-edited wheat plants with heritable edits [108]. Finally, compact base editors are being developed to reduce editor size and facilitate delivery through size-limited vectors, although their use in plant disease resistance breeding remains limited [109].

For PE, recent developments have improved both editor architecture and pegRNA design. Indeed, optimized pegRNA strategies showed that primer binding site length, with a melting temperature of approximately 30 °C was optimal, and reverse transcription template features strongly influence PE efficiency in rice [110]. Furthermore, computational tools such as PlantPegDesigner were developed to facilitate the rational design of efficient plant pegRNAs [111]. Engineered plant prime editors, such as ePPE, increased the efficiency of substitutions, insertions, and deletions by an average of 5.8-fold compared with the original PPE system [112]. Additional improvements were obtained through PEmax architectures and stabilized pegRNA expression, as shown by enpPE2, which strongly increased PE efficiency in rice [113]. More recently, ePPEplus was developed by introducing a V223A substitution into the reverse transcriptase domain of an ePPEmax*-derived architecture, markedly improving PE efficiency and enabling multiplex PE in regenerated hexaploid wheat plants [114]. Although BE and PE are not yet as widely applied as standard CRISPR-Cas-mediated knockouts, especially in plant disease resistance breeding, these advances show their potential as future platforms for precise and multiplex crop improvement. In addition to improving editing platforms themselves, advances in delivery systems are also essential to broaden the practical use of plant GE.

New emerging technologies, such as viral vectors, may hold the promise of overcoming the main limitations of applying plant GE for disease resistance breeding [58]. Plant viruses have been increasingly exploited as a delivery platform for CRISPR components and can be considered as an alternative to stable transformations. In particular, Virus-Induced Genome Editing (VIGE) has been developed as a tool to deliver CRISPR–Cas components through systemic infection [115]. In a study by Chen et al. [116], an engineered barley stripe mosaic virus (BSMV) was used in wheat plants overexpressing the Cas9 gene to deliver gRNAs targeting the Fusarium head blight susceptibility gene *TaHRC* [116]. Plants demonstrated a significant reduction in disease symptoms, and the authors identified heritable indels in progeny. Furthermore, using a TRV-based VIGE system targeting the eukaryotic elongation factor 1B gamma (*eEF1Bγ*) gene in *Nicotiana benthamiana*, Kang et al. [117] obtained plants with multiple homologs disrupted and observed reduced accumulation of tobacco etch virus (TEV). Recently, Lee et al. [118] developed a TRV-based VIGE system in tomato that delivers mobile guide RNAs systemically and enables high rates of heritable CRISPR-Cas9 mutations without additional tissue culture steps. While the above studies show that VIGE can produce targeted mutations in plant genomes with heritable effects, so far, only a few studies have been carried out to enhance plant disease resistance. However, the efficiency and demonstrated heritable capability of induced mutations could hold significant potential for functional silencing of plant S-genes [119]. Despite these strengths, some key limitations of VIGE are the inherent constraints of viral vectors themselves, such as the limited cargo capacity for delivering CRISPR components, challenges in achieving stable and high expression of editing cassette, and the restricted host range [120]. Very recently, transposon-derived GE platforms have emerged as promising alternatives for precise editing. These new platforms expanded nuclease-driven editing strategies and enabled DNA integration via cleavage mechanisms mediated by RNA-guided transposons. In the CRISPR-associated transposases (CASTs) system, Tn7-like transposons couple CRISPR elements, in particular the Cas12k in type V-K systems [121], with transposition proteins such as TnsB, TnsC, and TniQ, to guide DNA insertion without relying on host HDR [122,123]. Similarly, the obligate mobile element guided activity (OMEGA) systems encode more compact nucleases such as TnpB and IscB that use ωRNA guides to cleave DNA adjacent to Target Adjacent Motif (TAM) motifs, representing a distinct class of programmable editors with potential delivery advantages due to their smaller size [124,125,126]. Although these systems are not yet routine in plant GE for disease resistance, it has been demonstrated that the fusion of transposases and programmable nucleases has allowed the introgression of enhancer elements, open reading frame, and gene expression cassette in Arabidopsis and soybean with promising results [127]. In addition to transposon-associated systems, various next-generation CRISPR-derived platforms are further expanding the range of possibilities for plant GE modifications. One such approach is represented by retron-based systems, which enable the in vivo production of donor ssDNA that can be coupled to CRISPR components to support HDR, as demonstrated in *Nicotiana benthamiana* [128]. In parallel, newer targeted insertion platforms such as PrimeRoot have been developed to enable precise integration of large DNA fragments into plant genomes. PrimeRoot combines an enhanced plant prime editor with optimized pegRNA designs and recombinases, and has been reported to mediate targeted insertions of DNA fragments up to 11.1 kb; notably, it was also used to insert a PigmR cassette into rice, generating plants with increased blast resistance [129]. Additional other programmable insertion systems, including DNA polymerase-mediated GE such as DPET, as well as PASTE- and PASSIGE-related platforms, are further showing the rapid diversification of targeted DNA integration technologies, although their application to plant disease resistance breeding remains largely prospective so far [130].

It would also be important to mention that during the plant GE process, the introduction of selectable markers and editing cassettes is often required, but their persistence in the final plants is considered undesirable. To address this issue, site-specific recombination systems such as FLP/FRT and Cre/loxP could be used to remove unwanted DNA sequences and generate transgene-free lines. These systems use recombinase enzymes that bind short DNA target sites and mediate precise recombination between them, enabling the controlled removal or rearrangement of DNA sequences [131]. A key example was provided by Pompili et al. [132] who used a GE cassette integrating a CRISPR-Cas9 system to edit a fire blight S-gene in apple, and a FLP/FRT recombination system designed to remove the T-DNA harboring the expression cassettes for CRISPR-Cas9, the marker gene and the FLP itself. This allowed the generation of edited lines with reduced fire blight susceptibility and carrying a clean genetic background. Another example of this approach was provided by Orbegozo et al. [133], where a heat-inducible Cre/loxP system was used to excise the loxP sites flanking the selection marker gene (*nptII*) from transgenic potato plants. Therefore, these tools could be used as complementary during the plant GE process to contribute to addressing biosafety concerns by reducing the presence of exogenous DNA.

## 6. Precision Strategies for Plant Disease Resistance

**Figure 1:**
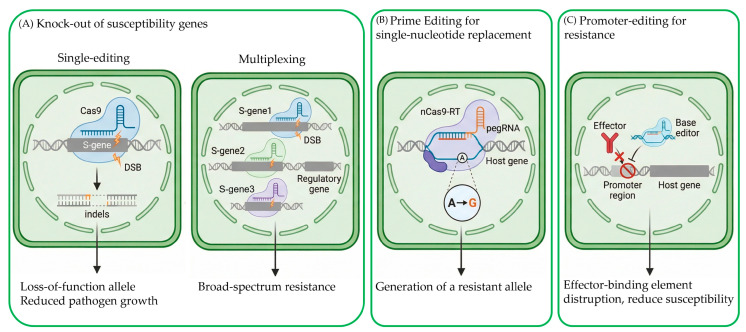
GE enables targeted modification of endogenous loci to reduce disease susceptibility and improve plant resistance. (**A**) CRISPR-Cas9 knockouts generate loss-of-function alleles in S-genes through double-strand breaks repaired by NHEJ, leading to indels and reduced pathogen compatibility. Multiplex CRISPR editing uses multiple guide RNAs to simultaneously edit different loci and generate broad-spectrum resistance. (**B**) Prime editing introduces precise nucleotide substitutions or small, defined sequence changes, allowing allelic replacement, mimicking natural resistant variants without requiring double-strand breaks or donor templates. (**C**) Target editing of promoter regulatory regions, such as EBEs, to prevent pathogen-induced activation of susceptibility genes while minimizing pleiotropic effects.

So far, gene knockouts via CRISPR-Cas system remain the most widely used strategy because the nucleases can generate loss-of-function alleles at predefined loci [58]. For example, CRISPR-Cas9 knockout of the tomato susceptibility loci *SlPelo* and *SlMlo1* produced elite tomato lines with reduced TYLCV accumulation and complete powdery mildew resistance [134,135]. It has been largely demonstrated that S-gene knockout worked similarly in other important crops, inducing broad-spectrum resistance to pathogens [136,137] (Figure 1). However, S-gene disruption does not always result in universally beneficial outcomes. Complete loss-of-function can lead to pleiotropic effects, as many genes involved in plant–pathogen interactions also play roles in essential physiological processes. Their disruption may therefore negatively affect plant growth, fitness, or responses to other pathogens. Notably, increasing evidence indicates that resistance gained against a specific pathogen may be accompanied by enhanced susceptibility to others [138]. Therefore, careful target selection and comprehensive phenotypic validation, including multi-pathogen assays, are essential steps to ensure the development of robust and agronomically viable resistant lines. For instance, in potato, the knockdown of *StNRL1* enhanced resistance to *Phytophthora infestans*, but simultaneously increased susceptibility to *Alternaria alternata*, indicating that the targeted locus can affect the whole pathosystem [138]. This contrasting result obtained after *StNRL1* editing shows that the outcome of gene knockout can vary depending on the pathogen. In this case, disruption of *StNRL1* improves resistance to *Phytophthora infestans* by preventing effector-mediated degradation of the immune regulator SWAP70, thereby enhancing defense-associated cell death. In contrast, the same modification increases susceptibility to *Alternaria alternata*, likely because necrotrophic pathogens can exploit host cell-death responses to promote infection. Therefore, beyond complete knockouts, other GE strategies such as allelic replacement and promoter editing have been developed [139] (Figure 1). In bacterial blight of rice caused by *Xanthomonas oryzae*, the susceptibility is caused by effector binding sites in the promoter region of the rice *Xa13* gene [140]. Accordingly, the introduction of specific mutations into the UPT box of *Xa13* using CRISPR-Cas12a technology abolished pathogen-induced expression and conferred resistance to rice blight. Interestingly, no negative agronomic phenotype, such as a decrease in fertility or seed-setting rate, was observed in the mutant lines [140]. To further increase precision at the nucleotide level, CRISPR-derived platforms such as BE and PE enable the engineering of resistance alleles and regulatory elements in plants [141] (Figure 1). In a recent study by Yu et al. [142], ScCas9-mediated PE was used to precisely perform three consecutive nucleotide substitutions on the endogenous *xa23* promoter in rice, restoring *Xa23* expression and broad-spectrum resistance to bacterial blight, without detectable pleiotropic effects. Another recent example has been provided in citrus, where the pathogen effector PthA4 of *Xanthomonas citri* can activate canker susceptibility by binding the EBE in the *LOB1* promoter. Performing a GE mediated by Cas12a-CBE, Jia et al. [143] converted cytosine to thymine on this EBE to generate transgene-free citrus plants with enhanced canker resistance. Furthermore, recent methodological improvements in PE have expanded its applicability in crops such as wheat, where optimized platforms enabled efficient multiplex nucleotide substitutions in regenerated hexaploid plants [114].

Beyond precise sequence modification, emerging GE strategies are increasingly inspired by the natural evolution of plant immune receptors. It is known that many plant R-genes evolved via integrated domain fusions in natural NB-LRR immune receptors [144]. Based on this concept, synthetic gene-fusion approaches are now being explored as a novel strategy to enhance disease resistance. For example, Kourelis et al. [145] engineered a chimeric immune receptor by fusing the Pik-1 NLR with a camelid nanobody able to recognize green fluorescent protein [145]. These synthetic receptors triggered immune responses when the corresponding target protein was present. Since nanobodies can be developed to bind different targets, NLR–nanobody fusions could potentially be adapted to recognize pathogen effectors delivered into plant cells. However, future works aiming to enhance plant disease resistance could aim to exploit endogenous domain fusions and recombination, inspired by naturally occurring integrated NLR architectures, to generate engineered receptors with novel recognition capacities [146].

## 7. Target Genes to Enhance Plant Disease Resistance Beyond Canonical R-Genes

**Figure 2:**
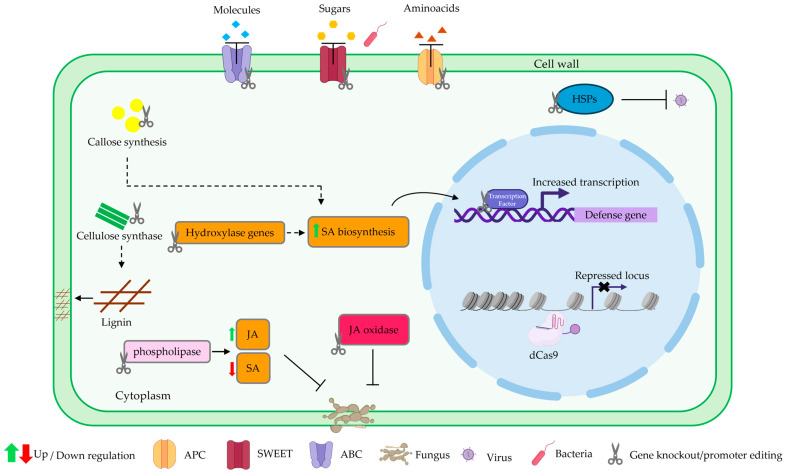
GE targets to enhance plant disease resistance. Schematic representation of a plant cell showing major host pathways that can be modified through GE to improve resistance to fungal, bacterial, and viral pathogens. Targeted modification of membrane transporters, cell wall biosynthesis genes, heat shock proteins, metabolic enzymes, hormone-related pathways, transcription factors, and epigenetic regulators can enhance resistance by limiting pathogen access to host resources, reinforcing structural barriers, or modulating immune signaling and defense gene expression. Solid arrows indicate direct interactions, dotted arrows represent multi-step or indirect pathways, and blunt-ended lines denote inhibitory effects associated with reduced disease susceptibility. Scissors indicate GE-mediated knockout/promoter editing.

Although resistance breeding has historically focused on R-genes, recent biotechnological approaches are increasingly targeting non-canonical host genes that influence plant disease response. These targets include TFs, HSPs, membrane transporters, and genes involved in hormone biosynthesis and signaling, cell wall organization, and metabolic pathways associated with pathogen susceptibility (Figure 2). Depending on the target, GE can enhance resistance by altering defense signaling, structural barriers, metabolic pathways, or pathogen access to host resources. By acting on these processes rather than directly on R-genes, precision breeding approaches can effectively improve plant disease resistance.

### 7.1. Transcription Factors

TFs are key players in physiological regulations [147]. Recent studies showed that in some cases, TFs can act as negative regulators of plant disease resistance, and GE can improve resistance by enhancing defense-associated genes, often with limited effects on yield (Figure 2). For instance, in rice, CRISPR-Cas9 knockouts of TF regulators have repeatedly increased resistance. The editing of an APETELA2/ethylene response factor (AP2/ERF) type TF *ERF922* boosted the resistance to blast, and improved resistance to many tested bacterial blight isolates, while maintaining key agronomic traits [148]. Similarly, CRISPR-Cas9 knocking out the AP2/ERF TF *OsERF104* reduced blast lesion number and damaged leaf area [149]. More recently, CRISPR-Cas9 mediating the loss-of-function of the TF *Bsrd1* was generated in japonica rice and associated with enhanced blast resistance [150]. The WRKY TFs provide another canonical example. CRISPR-Cas9-mediated knockout of *OsWRKY70* resulted in enhanced resistance to *Magnaporthe oryzae*, with a reduced lesion formation, lower fungal biomass, and limited invasive hyphal growth. This phenotype was associated with increased ROS accumulation and upregulation of defense-related genes, such as *OsPBZ1*, *OsPOX8.1*, *OsPOX22.3*, and *OsPR1b*. Transcriptomic analyses further revealed modulation of pathways related to plant–pathogen interaction, hormone signaling, and MAPK cascades. Notably, *OsWRKY70* was shown to bind the promoter of the JA-related regulator *OsbHLH6*, suggesting that its loss enhances resistance by releasing repression of JA-associated defense responses, although with trade-offs in cold tolerance [151]. Furthermore, with the attempt to provide broad-spectrum resistance, researchers silenced *OsWRKY36*, obtaining an increasing of lignin/sclerenchyma thickness and improved resistance to multiple rice planthoppers, as well as to the rice fungus *Magnaporthe oryzae*, while also supporting yield stability [152]. TF editing has also proven to be effective in other crops. For instance, in potato, dual-target CRISPR-Cas9 disruption of *StERF3* delayed disease progression and reduced pathogen biomass after *Phytophthora infestans* infections [60]. However, some studies highlighted that TFs do not always work as negative regulators. For example, a recent work of Chen et al. [153] showed that *SlWRKY75* overexpression increased resistance to *Ralstonia solanacearum*, whereas CRISPR-Cas9 knockout reduced resistance.

### 7.2. Heat Shock Proteins

For decades, HSPs have been recognized as hallmarks of the environmental stress response, and research on most HSPs has typically focused on their central roles in response to several abiotic stresses. However, different studies indicated that HSPs and molecular chaperones can function as susceptibility-associated host factors during pathogen infection [154] (Figure 2). Therefore, their silencing via GE is emerging as a strategy to enhance resistance in various crops. In *Nicotiana benthamiana*, Ge et al. [155] used CRISPR-Cas9 to generate *NbHsc70-2* knockout plants, demonstrating that the *Hsc70* chaperone contributes to PVY infection. Indeed, its disruption impairs viral protein stability and replication complex assembly, leading to reduced viral RNA accumulation, limited virus spread, and consequently enhanced resistance. In tomato, Qi et al. [156] used CRISPR-Cas9 to knockout *HSP40* (*SlDnaJ*) and found that edited T1 homozygous lines exhibited weaker TSWV symptoms and lower viral coat protein transcript levels. Indeed, they found that this modification altered the expression of key regulators in the salicylic acid (SA)/jasmonic acid (JA) signaling pathway with consequent changes in the plant immune response. In rice, Távora et al. [149] knocked out the chaperone gene *OsDjA2* with the CRISPR-Cas9 system and observed fewer blast lesions and reduced diseased leaf area in homozygous T1 mutants after *Pyricularia oryzae* infection. Together, these studies show that HSPs and molecular chaperones are not only involved in general stress responses but also play key roles in immune signaling and resistance pathways. Given their central regulatory functions in defense, targeting specific chaperone components through GE may provide a biological strategy to enhance durable disease resistance in crops [154,157].

### 7.3. Membrane Transporters and Carriers

Membrane transporters are key components of plant–pathogen interactions, as they control the flux of nutrients and ions that pathogens often exploit during infection [158]. Editing of genes encoding for transporters could restrict pathogen access to host resources and enhance plant resistance (Figure 2). Various studies observed that plant pathogens can use host sugar efflux for their own benefit. In aromatic rice, Zafar et al. [159] used CRISPR-Cas9 to disrupt four TALE EBEs in the *OsSWEET14* promoter, obtaining lines that showed an increased resistance to *Xanthomonas oryzae*. The EBEs disruption reduced bacterial blight susceptibility by preventing Xoo TALE-mediated activation of this sugar transporter, thereby limiting pathogen access to host-derived sugars required for infection. Similarly, a related study conducted by Zeng et al. [160] showed that editing the *OsSWEET14* promoter in a different cultivar had practical consequences for obtaining rice varieties with broad-spectrum resistance to Asian *Xanthomonas* spp.. Interestingly, plants showed an increased height and did not show a yield reduction. The SWEET activation induced by pathogens was investigated beyond rice bacterial blight. For example, Elliott et al. [161] targeted the cassava sugar transporter *MeSWEET10a*, which is induced by a Xanthomonas TAL effector (TAL20). Using CRISPR-Cas9, they generated cassava lines carrying mutations in the TAL20 EBE and within the *MeSWEET10a* coding sequence (CDS), showing that edited plants had reduced susceptibility to bacterial blight. The editing of the TAL20-binding region reduced bacterial blight susceptibility by preventing effector-mediated ectopic induction of this sugar transporter in leaves, likely limiting apoplastic sugar availability for pathogen proliferation while preserving endogenous gene function. Furthermore, the editing of the promoter region left an intact CDS, and thus a viable F1 progeny was recovered. In citrus, Khadgi et al. [162] similarly tested *SWEET10*, *SWEET12*, and *SWEET15* individually and in combination to evaluate how they affect bacterial disease development. Their findings showed that mutations in *SWEET15* reduce susceptibility to citrus canker across multiple cultivars. Interestingly, a recent experiment of Ponnurangan et al. [163] demonstrated that by editing the *OsSWEET11* promoter using the CRISPR-Cas9 system, edited lines displayed reduced susceptibility to *Rhizoctonia solani*, associated with reduced *OsSWEET11* induction, lower sucrose accumulation, and decreased fungal biomass during infection. Notably, they reported no major compromise of key agronomic traits in the majority of edited lines. Importantly, edits located outside the EBE region maintained normal growth and grain yield under nethouse conditions, whereas mutations overlapping the EBE resulted in reduced spikelet fertility and yield. These results highlight that the position of promoter edits is critical to achieve resistance without compromising agronomic performance.

Beyond sugar transport, editing of different membrane transporters can reduce host infections by limiting access to nutrients. A recent study in wheat, conducted by Javaid et al. [164], reported that CRISPR-Cas9-based knockout of *TaLr34*, an ABC transporter locus, induced enhanced leaf rust resistance without yield and agronomic performance penalties in elite wheat background. Notably, edited plants were evaluated under both glasshouse and field conditions across three growing seasons. The edited lines showed no significant differences in key agronomic traits, including plant height, spike characteristics, and grain yield, demonstrating that resistance can be achieved without detectable fitness penalties. In particular, a 3 bp deletion removed phenylalanine 546, mimicking the natural resistant Lr34res allele and likely modifying transporter activity and ABA-related signaling.

### 7.4. Cell Wall Biosynthesis

Several studies indicated that the modulation of cell wall biosynthesis genes can influence plant disease resistance [165]. As the primary structural barrier against pathogen invasion, the plant cell wall limits microbial penetration and functions as a dynamic signaling interface (Figure 2). For example, transgenic overexpression of genes involved in lignin biosynthesis has been associated with increased lignin deposition and improved resistance to fungal pathogens in several crop species [166]. Similarly, enhanced expression of callose synthase genes has been correlated with increased callose accumulation, restricting pathogen entrance [167]. However, recent GE studies have revealed that the relationship between cell wall biosynthesis and resistance is more complex than simple reinforcement. In some cases, targeted disruption of specific cell wall-related genes improved resistance. For instance, CRISPR-Cas9-mediated knockout of the tomato callose synthase *SlPMR4* reduced susceptibility to powdery mildew and late blight, via altered defense signaling and enhanced localized cell death responses [168]. Similarly, multiplexing of the wheat cellulose synthase gene *TaCESA7* resulted in cell wall remodeling, increased lignin deposition, and activation of defense-related pathways, leading to an enhanced resistance to stripe rust. Importantly, field evaluation showed no significant differences in major agronomic traits, including plant height, tiller number, spike length, thousand-grain weight, and grain size [169]. Despite the small number of available studies, modification of genes involved in the cell wall biosynthetic pathway could represent, in the future, valuable biotechnological alternatives for improving plant disease resistance as they contribute to both structural reinforcement and defense signaling [170].

### 7.5. Metabolic Enzymes and Hormone Biosynthesis

Metabolic enzymes are emerging as useful GE targets for resistance breeding (Figure 2). The editing of these loci can increase endogenous defense signaling and trigger plant broad-spectrum resistance. An example was provided in tomato where the disruption by CRISPR-Cas9 of *SlDMR6-1* and *SlDMR6-2*, which have hydroxylase activity and are involved in SA homeostasis, generated loss-of-function alleles that increased endogenous SA and produced broad-spectrum resistance across bacterial, oomycete, and fungal pathogens, with limited effects on plant growth under the tested conditions [171]. In potato, editing of *StDMR6-1* showed increased late blight resistance by impairing SA catabolism, which strengthens SA-associated defense responses without clear yield or tuber-quality penalties [172]. Notably, tubers also showed improved resistance to common scab, and plants displayed increased resistance to early blight [172]. A similar experiment was also conducted in banana, where CRISPR-Cas9 was used to target a MusaDMR6 ortholog, a susceptibility gene encoding 2-oxoglutarate Fe(II)-dependent oxygenase that is induced during compatible interactions, producing plants with enhanced resistance to Xanthomonas wilt in bioassays and greenhouse tests [173]. In rice, Liu et al. [174] used CRISPR-Cas9 to simultaneously mutate three SA 5-hydroxylase genes (*oss5h1*, *oss5h2*, and *oss5h3*), and reported stronger basal defense activation, with the upregulation of *OsWRKY45* and pathogenesis-related genes, and the triggering of broad-spectrum disease resistance. However, the silencing of the DMR6-like locus does not always have predictable effects on plant diseases. An example was provided by Wu et al. [175] who produced plants with a DMR6-like gene silenced (*OsF3H04g*) and noted a decrease in resistance to bacterial leaf streak, mainly due to the activation of expression of the complementary gene *OsS3H*. In perennial crops such as grapevine, a recent study by Djennane et al. [176] showed that editing *VvDMR6-1* by CRISPR-Cas9 reduced susceptibility to downy mildew (*Plasmopara viticola*), triggering higher SA levels and cis-resveratrol, but they also reported reduced growth and possible pleiotropic effects. Possible agronomic defects in editing DMR6 genes were also reported by a previous study [177], indicating the critical role of this gene in plant development.

Beyond SA, the editing of lipid- and JA-related genes is also emerging as a complementary route. In tomato, the knockout of the *SlPLC2* (phospholipase C2) gene mediated by the CRISPR-Cas9 system increased resistance to the necrotrophic fungus *Botrytis cinerea,* by decreasing reactive oxygen species (ROS) production that is required by the fungus for the infection [178]. In rice, multiplex CRISPR-Cas9 of JA oxidase (JAO) genes altered the JA metabolism and, when challenged with the blast fungus *Magnaporthe oryzae*, the mutant plants showed enhanced resistance [179]. However, mutants exhibited slightly reduced growth, fertility, and impaired seed filling [179]. Recent studies emphasized that the editing of S-genes could become an important strategy in breeding pipelines, but they must be paired with careful target choice and trait validation to manage possible off-targets and pleiotropic effects [58,180]. In this context, auxin plays a key role in coordinating growth and stress responses through interactions with other phytohormones, thereby influencing plant defense and adaptation to environmental conditions [181].

### 7.6. Epigenetic Regulators

CRISPR-based epigenetic editing tools can also modulate gene expression without altering the DNA sequence. Recent studies are demonstrating that genes involved in epigenetic regulation can represent promising non-classical targets for improving plant disease resistance, as they control chromatin accessibility and transcriptional activation of defensive signals [182] (Figure 2). For example, a recent experiment by Ge et al. [183] investigated the wheat DNA methyltransferase *TaMET1*. Their functional analyses showed that *TaMET1* acted as a negative regulator of resistance to powdery mildew. Silencing of the wheat DNA methyltransferase *TaMET1* led to reduced DNA methylation levels and promoted activation of the SA-related defense regulator *TaSARD1*, resulting in increased SA signaling and enhanced resistance. Very recently, Lin et al. [87] demonstrated that plant S-genes can be repressed by targeting epigenetic regulatory mechanisms using CRISPR-based tools. A nuclease-inactive dCas9-SunTag system engineered to target the promoters of the cassava S-genes *nCBP-1* and *nCBP-2* resulted in strong transcriptional repression through CRISPR interference, which enhanced plant resistance to the cassava brown streak disease. Chen et al. [184] demonstrated that in rice, the fungal effector Uv1809 promotes infection by interacting with histone deacetylase *OsSRT2*. The use of the CRISPR-Cas9 to silence the *OsSRT2* restored defensive gene expression and conferred broad-spectrum resistance to pathogens without detectable growth or yield penalties. While minimizing pleiotropic effects, the described approaches may allow a fine-tuning of epigenetic modification associated with resistance traits [185].

## 8. Technical and Practical Challenges

Despite the rapid expansion of GE technologies for plant disease resistance, different technical and practical challenges still limit their large-scale application in breeding programs. One of the main constraints in the obtainment of stable mutated plants is the variability in editing efficiency or sometimes the impossibility of transforming species and genotypes considered recalcitrant with standard transformation methods [186]. These issues significantly limit the practical use of CRISPR-based editing systems in various economically important crops [187]. The delivery methods influence the ability to regenerate edited plants from transformed cells. So far, Agrobacterium-mediated transformation and particle bombardment remain the most widely used delivery systems, although the genotypes used can influence the success rate of both methods [58]. Recent strategies such as viral vector-mediated delivery and ribonucleoproteins (RNPs) are being explored to overcome these limitations, although their application across diverse crop species is still limited and requires further optimization [58,115]. For example, the editing mediated by RNPs represents a relevant DNA-free strategy, as it relies on the direct delivery of preassembled Cas protein–gRNA complexes rather than DNA expression cassettes. RNP complexes can be introduced into plant cells through different delivery methods, such as PEG-mediated protoplast transfection, particle bombardment, and emerging nanoparticle-based systems. This approach can facilitate the recovery of transgene-free edited plants and may help reduce regulatory concerns associated with foreign DNA integration. Because RNPs act transiently, they may also reduce prolonged nuclease activity and the risk of unintended vector integration compared with DNA-based delivery systems. However, efficient RNP delivery remains technically challenging in many crops, as it still depends on the effective entry into plant cells and, in most cases, the regeneration from edited tissues or protoplasts. In parallel, nanoparticle delivery systems are emerging as a promising approach to transport nucleic acids and proteins into plant tissues. Because in some cases, nanomaterials can be used to protect biomolecular cargo and facilitate its movement across plant barriers, this strategy may help broaden GE applications to species or specific genotypes that are poorly compatible with Agrobacterium-mediated transformation or particle bombardment. Nevertheless, nanoparticle-mediated plant GE is still under development, and further optimization is required to improve cargo capacity, tissue penetration, delivery precision, scalability, reproducibility, and biosafety.

Another important technical concern relates to potential off-target mutations and genomic stability. Although CRISPR–Cas systems are generally considered highly specific, and the engineering of novel Cas variants is still improving efficiency and precision, unintended edits can occur when gRNAs bind to genomic regions with partial sequence similarity to target sequences [188]. The mutation of off-target sites may affect plant fitness, desired agronomic traits, and raise concerns about unintended genetic alterations in edited plants [58]. In general, plant GE generally raises fewer safety concerns than in animals because undesired somatic mutations transmitted to the offspring can be eliminated with conventional selection [189]. However, when CRISPR components are delivered through DNA vectors, unintended insertion of vector-derived sequences may occur at on or off-target sites, which can classify edited plants as transgenics [190]. Several approaches have been developed to mitigate this issue, including improved gRNA design algorithms, high-fidelity Cas variants, and editing platforms that avoid double-strand DNA breaks [70]. Nevertheless, an accurate detection of off-target mutations is crucial and could be technically challenging in crops with large and complex genomes [191]. Furthermore, crops could have multiple homolog gene copies, which can mask the effect of a single-gene edit. Therefore, achieving the desired phenotype often requires the simultaneous editing of homologs through multiplexing strategies [192,193]. However, while multiplexing can target multiple loci simultaneously, editing efficiency tends to decrease as the number of targets increases, making it difficult to obtain complete knockout lines in highly polyploid genomes. Furthermore, multiplexing could increase the risk of pleiotropic effects, as several interconnected pathways are modified at the same time. Therefore, careful target selection and phenotypic evaluation remain essential. In the future, advances in editing technologies are expected to overcome genome redundancy and facilitate the generation of complete knockouts even in highly polyploid crops [194].

Beyond technical limitations, regulatory and social factors represent major practical challenges for the practical utilization of GE-modified crops. Regulatory frameworks for GE crops differ considerably across regions. In the European Union, edited plants were initially regulated under the same legislation as genetically modified organisms (GMOs) following the 2018 ruling of the Court of Justice of the EU under Directive 2001/18/EC. However, recent policy developments have moved toward a differentiated regulatory framework for plants produced through new genomic techniques (NGTs), distinguishing Category 1 NGT plants, considered equivalent to conventional varieties, from Category 2 NGT plants, which remain subject to existing GMO legislation [195]. Other important aspects to mention for the future deployments of GE in crop breeding are related to intellectual property and biosafety issues. Recent analyses indicate that access may be restricted by intellectual property rights on CRISPR technologies themselves, as well as patents on gene-edited traits or plant varieties, which together may create uncertainty and limit freedom to operate, particularly for public and small-scale breeding programs. In parallel, recent articles focused on biosafety issues, stating that edited plants should be assessed case by case, taking into account both the effects of the modified traits and the potential unintended changes, such as off-target modifications, potential integration and persistence of editing components, gene flow, and ecological interactions after the release of modified plants [196,197,198].

Beyond these regulatory and governance challenges, the practical application of edited plants in the field remains complex. Resistance traits identified under controlled conditions may behave differently in agricultural environments, where plants are exposed to multiple biotic and abiotic stresses simultaneously [4]. Consequently, edited lines require extensive phenotypic validation across diverse field conditions before being integrated into breeding programs [2]. Although GE offers unprecedented opportunities for precision breeding, continued improvements in delivery systems, editing accuracy, regulatory acceptance, and field validation are necessary before its full optimization for developing disease-resistant crops.

## 9. Conclusions and Future Perspectives

Biotechnological tools are rapidly shaping new strategies for improving plant disease resistance. While conventional breeding has produced many resistant cultivars, it is often limited by long breeding cycles and the availability of useful genetic diversity. GE technologies now provide a faster and more precise way to introduce resistance traits by directly modifying endogenous plant genes. In recent years, CRISPR-based systems have been widely used to disrupt S-genes, engineer resistance alleles, and modify regulatory regions involved in plant–pathogen interactions. As discussed in this review, GE approaches have already targeted multiple classes of genes beyond classical R-genes, including TFs, HSPs, membrane transporters, cell wall biosynthesis components, metabolic enzymes, and epigenetic regulators. Analyzed studies showed that the modification of host processes could provide effective resistance while preserving key agronomic traits. Another important advantage of GE is the possibility of combining different targets to generate more durable resistance. Multiplex editing allows the simultaneous modification of different loci, which can help in obtaining broad-spectrum resistance. In addition, newer tools enable targeted insertions and precise nucleotide changes that mimic natural mutations, reducing the need for complete gene knockouts and potentially minimizing pleiotropic effects.

A major challenge for GE-based precision breeding is the occurrence of pleiotropic effects associated with the modification of S-genes, which often play roles in essential physiological processes. In some cases, resistance to a specific pathogen may be accompanied by increased susceptibility to others, reflecting the interconnected nature of plant immune networks. Therefore, future strategies will require the optimization of target selection and systematic multi-pathogen validation to ensure durable and broadly effective resistance traits. As editing tools become more efficient, these strategies are expected to become increasingly integrated into crop breeding pipelines. In parallel, emerging high-resolution approaches such as single-cell and spatial transcriptomics are providing new insights into cell-type-specific and spatially occurring stress responses. These technologies enable the identification of key regulatory genes and pathways that may be masked in bulk analyses, offering new opportunities to refine target selection and improve the precision of GE strategies for durable disease resistance [199].

Looking forward, the integration of multi-omics approaches, including transcriptomics, proteomics, and the previously mentioned single-cell and spatial transcriptomics technologies, is expected to further refine target identification and support the design of optimal gene combinations for multiplex GE aimed at durable and broad-spectrum resistance. Furthermore, emerging genome engineering platforms are expected to expand the range of approaches available for improving plant disease management. For instance, CRISPR-based gene drive systems have been proposed as a complementary strategy to control plant diseases by targeting insect pests and pathogen vectors. By promoting the spread of engineered traits within populations, these systems could reduce vector abundance or limit pathogen transmission. However, their effectiveness could be highly species-specific, and their application in crop protection remains largely prospective, with important ecological and biosafety considerations [200,201,202].

Therefore, continued improvements in GE technologies, together with a deeper understanding of plant immunity, are expected to accelerate the development of disease-resistant crops and strengthen the role of precision breeding for sustainable agriculture.

## Figures and Tables

**Table 1:** Timeline from conventional breeding to modern approaches in plant breeding for disease resistance.

Breeding Approach	Period	Key Features and Milestones	Costs Range *	Time Range (Years) *
Conventional	Early 1900s–Present	Phenotypic selection and crossing with wild relatives; development of resistant cultivars based on natural variation	Low–Medium (mainly field trials, labor, phenotyping)	6–12+
Early precision breeding (MAS, QTLs, genomic selection)	1990s–Present	Introduction of molecular markers (MAS), QTL mapping and pyramiding (1990s–2000s), and genomic selection (2010s) to improve selection accuracy	Medium (genotyping and modeling costs scale with population size)	2–8
Transgenic approaches (overexpression, RNAi, VIGS)	Late 1990s–Present	Stable genetic transformation enabling gene overexpression and RNA interference; first GM crops (1990s) and RNAi-based resistance strategies (2000s)	Medium–High (transformation and event validation)	3–6
Genome editing	~2000–Present	Programmable nucleases (meganucleases, ZFNs, TALENs) followed by CRISPR systems (early 2010s) enabling efficient and multiplex plant genome editing; development of base and prime editing (late 2010s–early 2020s)	Low–Medium	1–4 (shorter in model crops, optimized or fast-generation systems)
Emerging precise editing	~2020–Present	Novel nucleases, expansion of CRISPR toolbox for transcriptional regulation (CRISPRa/i), RNA targeting (Cas13), targeted DNA insertion systems (e.g., PrimeRoot, CASTs) and retron-based systems supporting template-directed editing, advanced delivery platforms (e.g., viral vectors), and use of complementary strategies for marker excision (e.g., FLP/FRT, Cre/loxP)	Medium (high variable, technologies are still under optimization)	2–5+ (highly variable)

* Costs and time ranges refer to the generation of improved lines. These comparisons do not consider cultivar registration, regulatory approval, commercialization, or seed release. Ranges may vary widely depending on crop species, genotype, transformation/regeneration efficiency, infrastructure, and validation path.

## Data Availability

No new data were created or analyzed in this study. Data sharing is not applicable to this article.

## Abbreviations

The following abbreviations are used in this manuscript:

ABE	Adenine base editor
CASTs	CRISPR-associated transposases
CBE	Cytidine base editor
CDS	Coding sequence
EBE	Effector binding element
GE	Genome editing
GMOs	Genetically modified organisms
HDR	Homology-directed repair
HSPs	Heat shock proteins
JA	Jasmonic acid
MAS	Marker-assisted selection
NB-LRR	Nucleotide-binding leucine-rich repeat
NHEJ	Non-homologous end joining
NGTs	New genomic techniques
OMEGA	Obligate mobile element guided activity
PAM	Protospacer-adjacent motif
QTL	Quantitative trait loci
R-gene	Resistance gene
RNAi	RNA interference
RNPs	Ribonucleoproteins
ROS	Reactive oxygen species
SA	Salicylic acid
S-gene	Susceptibility gene
sgRNA	Single-guide RNA
ssDNA	Single-strand DNA
TALE	Transcription activator-like effector
TALEN	Transcription activator-like effector nucleases
TAM	Target adjacent motif
TF	Transcription factors
VIGE	Virus-induced genome editing
VIGS	Virus-induced gene silencing
ZFNs	Zinc-finger nucleases
